# Upregulation of EMID1 Accelerates to a Favorable Prognosis and Immune Infiltration in Lung Adenocarcinoma

**DOI:** 10.1155/2022/5185202

**Published:** 2022-09-19

**Authors:** Yufeng Shao, Zhong Zheng, Sen Li, Guangyu Yang, Fuwei Qi, Fan Fei

**Affiliations:** The First People's Hospital of Taicang City, Taicang Affiliated Hospital of Soochow University, Suzhou, China

## Abstract

Lung cancer is a difficult-to-treat cancer. Lung adenocarcinoma (LUAD) is the main subtype of lung cancer. Although there are many ways to treat lung cancer, the survival rate of patients is low. Therefore, novel molecules need to be identified to diagnose and treat LUAD. This study utilized The Cancer Genome Atlas (TCGA) LUAD data to analyze and validate the value of EMID1 as a LUAD diagnostic surface marker and overall survival prognostic marker. Differential expression analysis formally confirmed that decreased EMID1 expression was significantly associated with advanced stage and metastasis of lung cancer. Kaplan–Meier survival analysis showed that the patients with low EMID expression are dismal. The relationship between clinicopathological features and EMID1 was scored using Wilcoxon signed-rank test and R (v.3.5.1) logistic regression and suggested that patients with low EMID1 expression had a worse prognosis than patients with high EMID1 expression. (Gene Ontology) GO, Kyoto Encyclopedia of Genes and Genomes(KEGG), and gene set enrichment analysis (GSEA) were performed to investigate the potential mechanism of EMID1 expression on the prognosis of LUAD and suggested that Notch signaling pathway may be an important biological pathway for EMID1 to play a role in LUAD. Further, combined with univariate and multivariate Cox regression analysis, it was speculated that high and low levels of EMID1 expression and the logistic regression analysis of related clinical variables had significant clinical significance to verify the underlying mechanism of LUAD focus and prognosis. EMID1 plays an important role in the immune milieu of LUAD. Meanwhile, the correlation between tumor-infiltrating immune cells and genes was assessed using CIBERSORT, and it was found that the level of B cell infiltration was positively correlated with the expression of EMID1, all of which were validated in the GEO and GEPIA databases. In all, this study helps to understand the immune microenvironment of LUAD and improve the survival of patients with LUAD. Thus, EMID1 may be a novel immune-related prognostic marker of LUAD.

## 1. Introduction

Lung cancer, a kind of refractory cancer, is the main cause of cancer-related deaths [1, 2]. It has the lowest five-year survival rate among some major cancers, such as colon cancer, breast cancer, and prostate cancer [3, 4]. According to histology, non-small cell lung cancer (NSCLC) is one of the main subtypes of lung cancer and accounts for approximately 85% of all lung cancer cases. NSCLC can be divided into three types: squamous cell carcinoma, adenocarcinoma, and large cell carcinoma [5, 6]. Lung adenocarcinoma (LUAD) is the most common type of lung cancer and accounts for about 40% of all lung cancers. LUAD develops from small airway epithelial type II alveolar cells that secrete mucus and other substances [7–9]. At present, the treatment methods of lung cancer mainly include surgery, radiotherapy, chemotherapy, targeted cancer therapy, and immunotherapy. However, the survival rate of patients has not improved and remains at 15% within five years of treatment [10]. Therefore, it is essential to urgently identify new molecules for the treatment and diagnosis of LUAD to improve the survival of patients with LUAD.

The environment of tumor growth is called tumor microenvironment (TME), which consists of blood vessels, lymphatic vessels, extracellular matrix, immune cells, stromal cells, secretory proteins, RNA, and small organelles [11]. TME plays an important role in tumor occurrence, development, metastasis, recurrence, and drug resistance. Immune cells are an important part of the TME. Previous studies showed that immune cells play an indispensable role in tumor development. For instance, regulatory T cells (Tregs) can produce IL-10, transforming growth factor-*β* (TGF-*β*), and cell-mediated cell contact (CTLA4) to exert an immunosuppressive function and inhibit the recognition and clearance of tumor cells by the immune system [12–14]. In addition, high expression of Tregs in TME has been shown to be associated with poor prognosis in some cancers, for example, breast cancer, hepatocellular carcinoma, kidney renal clear cell carcinoma, and pancreatic ductal adenocarcinoma [15–19]. On the contrary, Tregs have been proved to be related to the good prognosis of Hodgkin's lymphoma by directly inhibiting the growth of tumor cells [20–23]. Most B cells exist at the edge of tumor invasion. Some studies have found that B cell infiltration in TME is related to good prognosis of some cancers [24, 25]. However, the role of immune cells in TME of LUAD is not clear.

Emilin (elastin microfibril interphase located protein) is a juxtaposition protein consisting of four protein domains: a short collagenous stalk, a self-interacting globular C1q domain at the C-terminal, an extended region of potential helical coil structure, and a cysteine-rich domain at the N-terminal (EMI domain) [26]. Larson et al. showed that EMID1 is associated with Pca bone metastasis, since it is highly expressed in osteoblasts [27]. However, no study to date has investigated the role of EMID1 in the development of cancer. Therefore, this study aims to evaluate the prognostic value of EMID1 expression in human LUAD.

## 2. Materials and Methods

### 2.1. Data Acquisition

This study identified and downloaded an open dataset containing gene expression profiles and prognosis information of tumor and normal tissues from TCGA (https://portal.gdc.cancer.gov/), including 535 tumor samples and 59 normal tissues. Then, 522 clinical data were used for clinical correlation analysis, and 494 patients with full clinical information were included for survival analysis. To study the effect of EMID1 expression on TME, 535 tumor tissues were used for CIBERSORT analysis.

### 2.2. Construction of PPI Network and Screening of Hub Genes

Differentially expressed genes (DEGs) based on EMID1 expression levels were submitted to the STRING database for the construction of protein-protein interaction (PPI) network. Cytoscape (version 3.7.1) was used to analyze the PPI networks, with a composite score >0.4 as cutoff. The Cytoscape plugin cytoHubba was used to screen the top 10 hub genes, and then MCODE was used to perform molecular complex detection to obtain modules.

### 2.3. Correlation of EMID1 Expression with Survival Prognosis and Clinical Features

Cox proportional hazards model and Kaplan–Meier plotter analysis were used to evaluate the association of EMID1 expression with overall survival and various clinical variables. EMID1 expression was correlated with clinicopathological features, including age, sex, tumor grade, and stage (T: tumor status, N: lymph node, M: distant metastasis).

### 2.4. Logistic Regression of Clinicopathological Features Based on EMID1 Expression Level

Variables with a *P* value < 0.05 in the multivariate analysis were included in the prognostic model. The performance and discriminative ability were assessed using Harrell's concordance index. Nomograms were constructed to predict the 3-year, 5-year, and 10-year survival rates of patients with SKCM based on predictive models with identified prognostic factors. Calibration was defined as a prediction from the nomogram compared with the observed outcomes.

### 2.5. Gene Set Enrichment Analysis (GSEA)

We analyzed GO item and KEGG pathway with GSEA to explore the possible biological functions of EMID1 in LUAD. In the enrichment results, a false discovery rate (FDR <0.25) and the nominal *P* value (*P* < 0.05) were considered statistically significant.

### 2.6. Assessment of Tumor-Infiltrating Immune Cells in LUAD

In this study, we aimed to determine the proportion of 22 kinds of immune cells in LUAD by CIBERSORT to evaluate their correlation with survival rate and molecular subsets. To evaluate the effect of EMID1 expression, we uploaded the gene expression data of 535 samples obtained from TCGA on the CIBERSORT portal. The algorithm uses 1000 default signature matrices, estimates the *P* value of deconvolution through Monte Carlo sampling, and establishes the confidence of the results. According to *P* < 0.05, the immune cells that may be affected by EMID1 were selected. In addition, correlation thermography was used to detect the correlation of 22 immune cells. Additionally, we used TIMER to explore the collection of EMID1 expression and immune infiltration level in LUAD and to explore the cumulative survival in LUAD.

### 2.7. Verification Analysis

GSE8894 dataset was obtained from Gene Expression Omnibus (GEO) database and contains clinical information of 61 samples, which were used for survival analysis. GEPIA is an online database that uses standard processing flow to analyze 8,587 normal and 9,736 tumor samples in GTEX and TCGA [28]. We used the survival module of GEPIA to analyze the relationship between the prognosis of patients with LUAD and the expression of EMID1. The differential expression of EMID1 between tumor and normal tissues was observed by boxplot, and the differential expression of EMID1 in different pathological stages was compared.

### 2.8. Statistical Analysis

R version 3.5.1 was used for statistical analysis. The Wilcoxon signed-rank test, along with a logistic regression, helped evaluate the correlation of clinic-pathological features with EMID1. The correlation between tumor-infiltrating immune cells and genes was assessed by CIBERSORT. A *P* value < 0.05 in all tests was regarded statistically significant.

## 3. Results

### 3.1. Differential Expression Analysis of EMID1 in TCGA-LUAD

**Figure 1:**
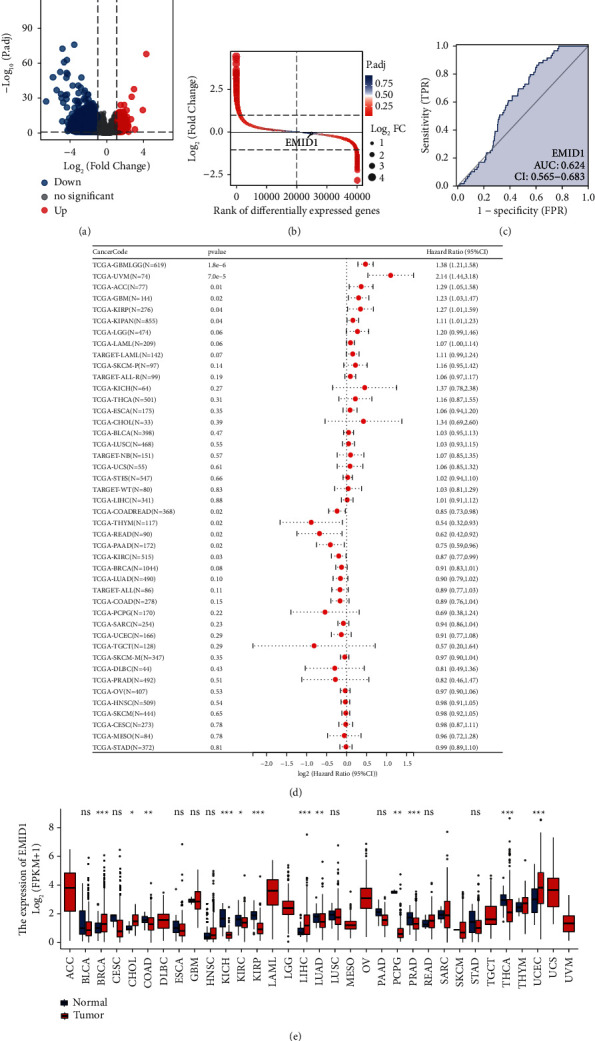
Differential expression analysis of EMID1. (a) The median expression of EMID1 was divided into high and low expression groups, and the differential gene volcano plot between groups. (b) The differential ranking plot showed that the threshold of TCGA-LUAD was |log2(FC)|>1 and p.adj < 0.05, the number of DEGs meeting this threshold is 1229, of which 945 are of high expression (logFC is positive), and 284 are of low expression (logFC is negative). The figure indicates the difference in the expression of EMID1. (c) ROC curve of EMID1 between normal samples and LUAD samples. (d) Forest plot of the effect of EMID1 in TCGA pan-cancer. (e) EMID1 in TCGA Boxplots of expression differences in pan cancer.

To explore the mRNA expression of EMID1 in normal human tissues, we combined GTEX and TCGA-LUAD datasets to study the expression of EMID1 in tumor tissues. We divided the tumor samples into high-andlow-expression groups based on the median expression of EMID1. We then obtained the co-expressed genes of EMID1 by difference analysis between the groups and displayed the gene difference volcano plot (Figure 1(a)). At the same time, the difference ranking map (Figure 1(b)) showed that the threshold of TCGA-LUAD was |log2(FC)| > 1 and *P* adj <0.05, and the number of DEGs satisfying this threshold was 1,229. Of these, 945 were upregulated and 284 were downregulated. We assessed the diagnostic efficacy of EMID1 in TCGA-LUAD to discriminate between normal and LUAD samples by ROC curve, AUC: 0.624 (95%CI: 0.565 − 0.683) (Figure 1(c)). To further analyze the effect of EMID1 on pan-cancer, we used a forest plot to display the effects of high expression of EMID1 on the risk of various tumors. 74) 7.0e-5 2.14 (1.44, 3.18), ACC (N = 77) 0.01 1.29 (1.05, 1.58), GBM (N = 144) 0.02 1.23 (1.03, 1.47), KIRP (N = 276) 0.04 1.27(1.01, 1.59), and KIPAN (N = 855) 0.04 1.11 (1.01, 1.23) were statistically significant (Figure 1(d)). Subsequently, we compared the differences in EMID1 expression in TCGA pan-cancer with boxplots, which were statistically significant in tumor types including BLCA, CESC, CHOL, KICH, KIRC, KIRP, LIHC, LUAD, PAAD, PCPG, THCA, and UCEC academic significance (Figure 1(e)).

### 3.2. Construction and Enrichment Analysis of PPI Network of EMID1 DEGs

**Figure 2:**
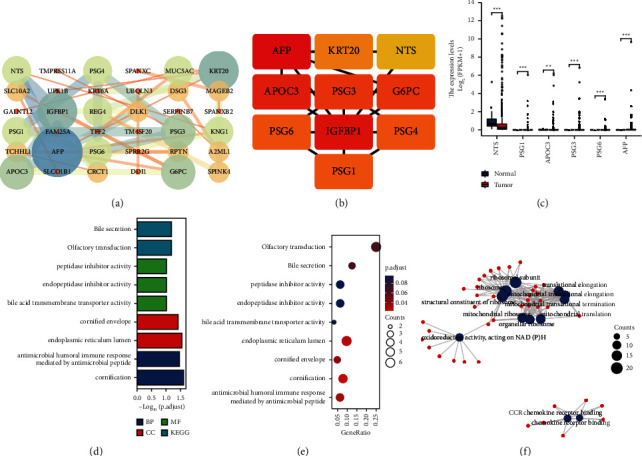
Construction and enrichment analysis of PPI network of co-expressed differential genes of EMID1. (a) PPI network of differentially co-expressed genes of EMID1. (b) Top10 Hub genes that are closely related among differentially co-expressed genes of EMID1 analyzed by CytoHubba. (c) Bins of differentially expressed Hub genes between TCGA-LUAD normal samples and LUAD samples. (d–f) GO and KEGG enrichment analysis of differentially expressed co-expressed genes of EMID1 in bar plot, bubble plot and chord plot.

We first obtained the correlation of EMID1 differentially co-expressed genes through the STRING database and constructed a PPI network (Figure 2(a)). To further screen the co-expressed genes closely related to EMID1, we analyzed the closely related top 10 hub genes among the differentially co-expressed genes of EMID1 by cytoHubba (Figure 2(b)). We showed differential expression between TCGA-LUAD normal samples and LUAD samples. The expression of hub genes in LUAD was significantly different from that in the normal samples (Figure 2(c)). The subsequent enrichment analysis of GO and KEGG pathways of differentially expressed co-expressed genes of EMID1 are displayed in bar graphs, bubble charts, and chord graphs. It was found that EMID1 DEGs were mainly enriched in antibacterial body fluids, immune response mediation, endoplasmic reticulum lumen, multiple enzyme inhibitor activities, and bile acid secretion (Figure 2(d)-2(f).

### 3.3. GSEA Helps to Identify EMID1 Linked Signaling Pathways

**Figure 3:**
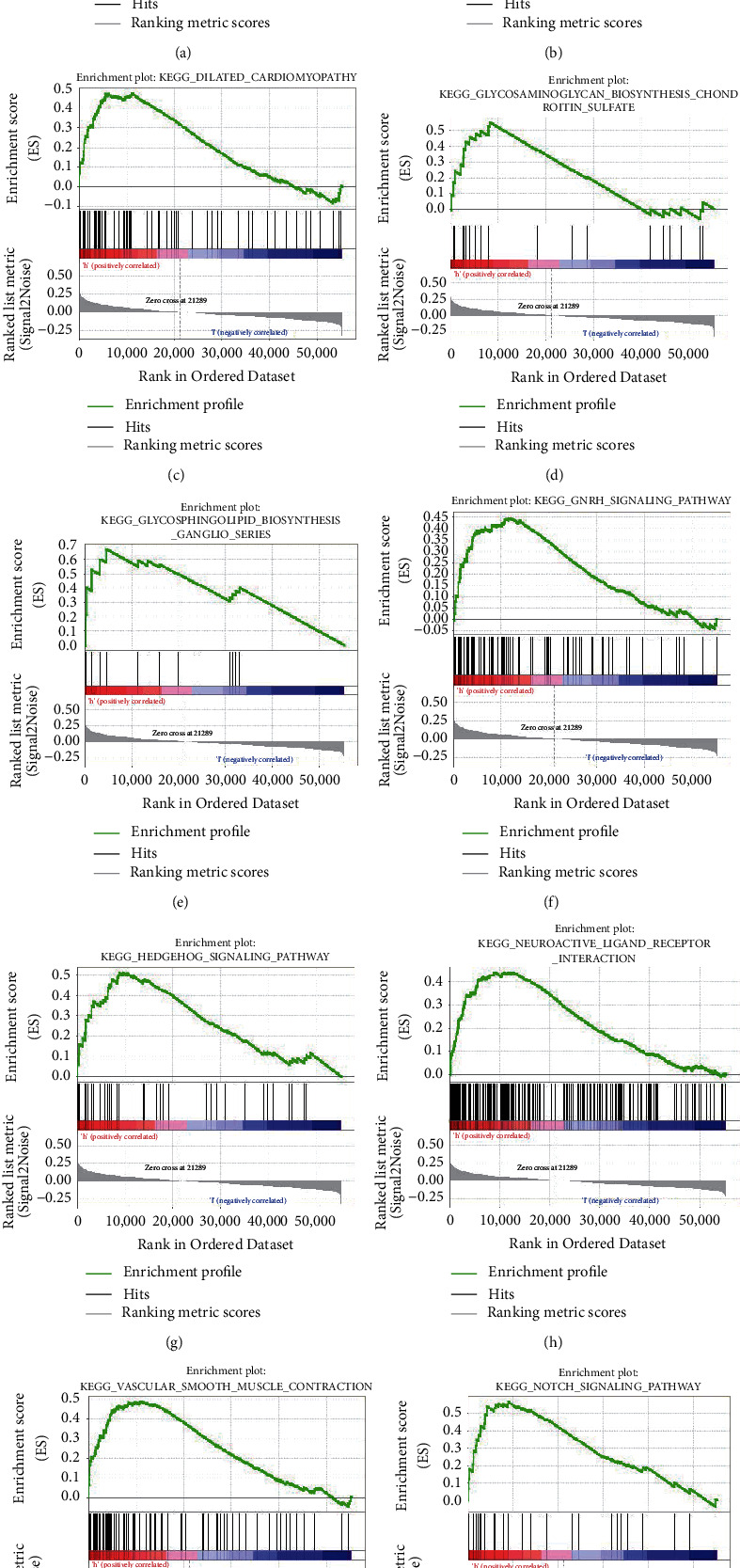
Gene set enrichment analysis (GSEA). The results show melanogenesis (a), basal cell carcinoma (b), dilated cardiomyopathy (c), glycosaminoglycan biosynthesis heparin sulfate (d), glycosphingolipid biosynthesis ganglio series (e), GnRH signaling pathway (f), hedgehog signaling pathway (g), neuroactive ligand-receptor interaction (h), vascular smooth muscle contraction (i), and notch signaling pathway (j) are differentially enriched in high EMID1 expression. ES, enrichment score; NES, normalized ES; NOM p-val, normalized *P*-value.

In this study, GSEA was performed between low-andhigh-expression groups of EMID1 to determine the signal pathways significantly related to EMID1 in LUAD (Table 1).  Figure 3 shows that 10 KEGG pathways were associated with a high-expression phenotype of EMID1, including melanogenesis, basal cell carcinoma, vasoconstriction, glycosaminoglycan biosynthesis of heparin sulfate, Notch signaling pathway, neuroactive ligand-receptor interaction, Hedgehog signaling pathway, ganglioside biosynthesis series, GnRH signaling pathway, and dilated heart myopathy.

### 3.4. Association of EMID1 Expression with Clinicopathological Variables and Survival Outcomes

**Figure 4:**
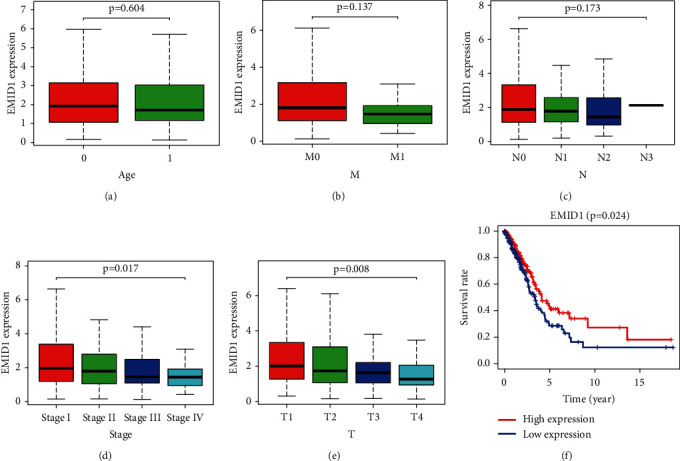
(a–e). Association with EMID1 expression and clinicopathologic characteristics, including A: Age, B: M, C: N, D: stage, E: T, T: tumor status, N: lymph node, M: distant metastasis. (f). The relationship between the expression of EMID1 and the prognosis of patients.

We obtained the clinical and gene expression data of 522 samples from TCGA and 61 samples from GEO database. Specific patient characteristics of LUAD are shown in Table 2. We evaluated EMID1 expression data from TCGA. As shown in Figures 4(a)–4(e), the decreased expression of EMID1 was significantly correlated with clinical stage (*P*=0.017) and tumor status (*P*=0.008). According to logistic regression analysis, the median expression of the dependent variable of EMID1 expression classification was 2.5, indicating a poor prognosis (Table 3). In patients with LUAD, the decreased expression of EMID1 was significantly correlated with clinical stage (stage III vs. stage I, *P*=0.012; stage IV vs. stage I, *P*=0.009), tumor status (T3 vs. T1, *P*=0.041), lymph node (N2 vs. N0, *P*=0.007), and distant metastasis (M1 vs. M0, *P*=0.030). Therefore, compared with the high-expression group, patients with low EMID1 expression had a higher risk of developing lung cancer. Moreover, Kaplan–Meier survival analysis also suggested a poor prognosis in low EMID1 expression LUAD, with *P*=0.024 (Figure 4(f)).

### 3.5. Verification Analysis of EMID1

**Figure 5:**
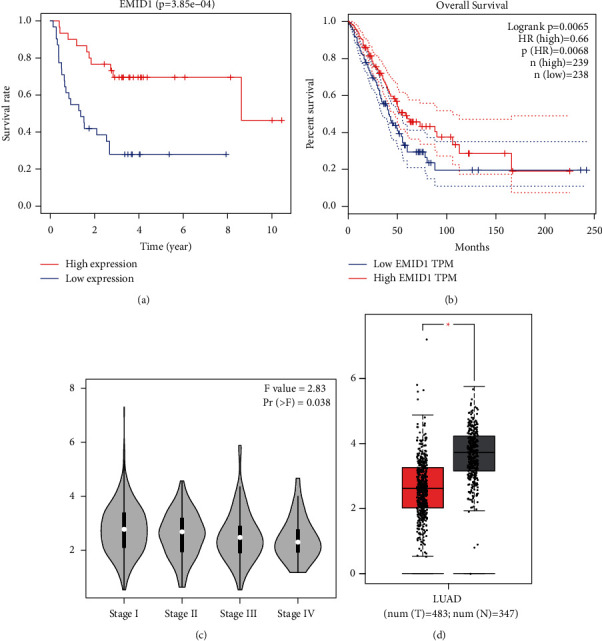
(a) The relationship between the expression of EMID1 and the prognosis of LUAD patients in GEO database. (b) The expression level and overall survival rate of EMID1 in GEPIA database. (c) The expression of EMID1 was different in different pathological stages. (d) The expression level of EMID1 in normal and cancer tissues.

As shown in Figure 5(a), Kaplan–Meier survival analysis showed that the prognosis of patients with high EMID1 expression was better than that of patients with low EMID1 expression (*P* < 0.001). At the same time, we found that low expression of EMID1 was significantly related with low OS (*P* < 0.001) and late stage of pathology through the GEPIA database (Figures 5(b)-5(c)). The expression of EMID1 in tumor tissues was significantly lower than that in normal tissues (Figure 5(d)).

### 3.6. Clinical Correlation Analysis of EMID1 with OS Prognosis of LUAD

**Figure 6:**
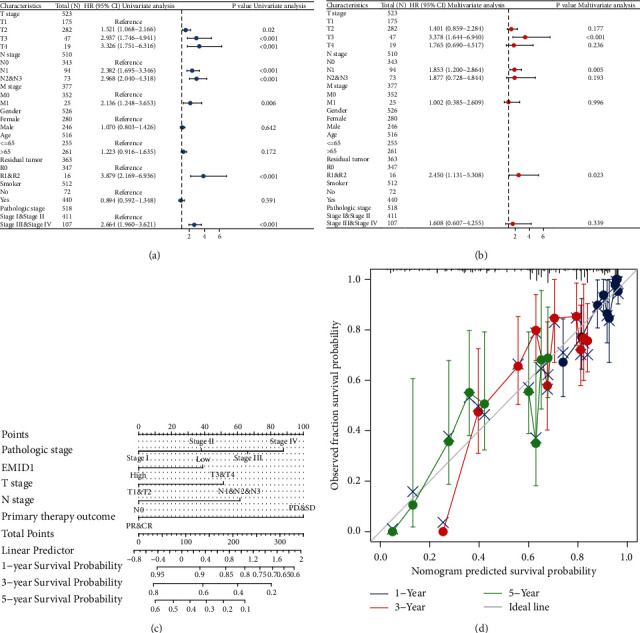
Clinical correlation analysis of EMID1 (a-b) Forest plot of univariate and multivariate Cox regression analysis of EMID1 in TCGA-LUAD; (c) nomogram of clinical correlation analysis of EMID1 in overall survival prognosis in LUAD; (d) calibration curve of EMID1 for LUAD1, 3, and 5-year overall survival prognosis.

Clinicopathological data were obtained from TCGA, and we analyzed the prognostic risk of key clinical variables of LUAD. The results of univariate and multivariate Cox regression analysis in TCGA-LUAD for the stratified variables of clinicopathological characteristics of OS in TCGA were plotted (Figures 6(a)-6(b), Table 4). The Cox regression analysis suggested that residual tumor, high and low expression of EMID1, tumor stage, and primary therapy outcome affect the OS of LUAD. The nomogram shows the effect of EMID1 on 1-, 3-, and 5-year OS prognosis of LUAD. Nomogram of clinical correlation analysis of EMID1 showed the overall survival status in LUAD (Figure 6(c)). At the same time calibration curve of EMID1 for LUAD1, 3, and 5-year overall survival prognosis were shown in Figure 6(d).

### 3.7. Correlation of EMID1 Expression with TIICs and Immune-Related Biomarkers

**Figure 7:**
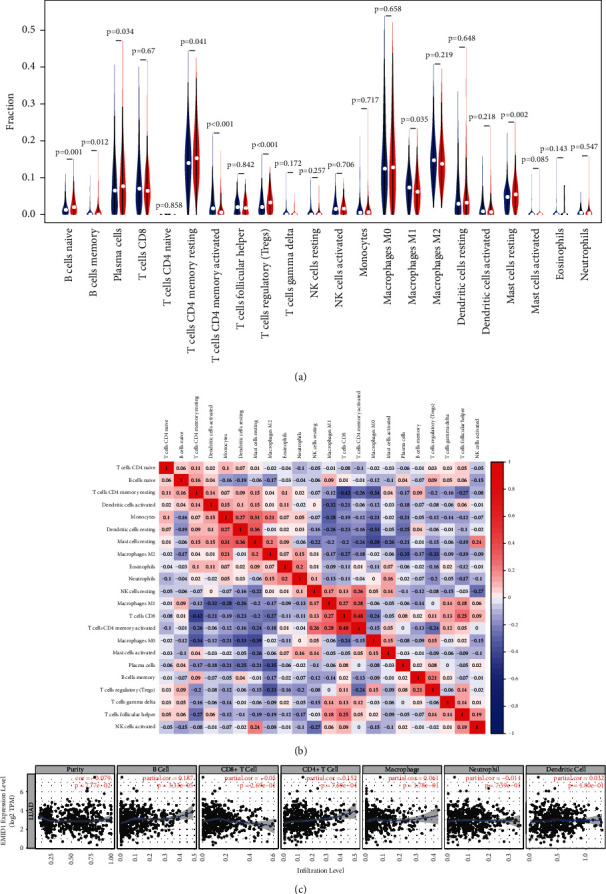
(a) The relative proportion of 22 kinds of immune cell infiltration of lung adenocarcinoma in high-EMID1 expression group and low EMID1 expression group were estimated by CIBERSORT. (b) The correlation of different proportion of infiltrating immune cell subsets in lung adenocarcinoma. (c) The correlation of immune cell infraction and EMID1 expressions.

To study whether the expression of EMID1 affects the immune microenvironment of LUAD, the gene expression profiles of the samples were analyzed using the CIBERSORT algorithm to evaluate the density of 22 immune cells in LUAD. First, according to the expression of EMID1, 535 tumor samples were divided into two types: 267 cases of low expression and 268 cases of high expression. Then, the relative proportion of 22 immune cells in these tumor samples was estimated by CIBERSORT. The results are shown in Figure 7(a). Naïve B cells (*P*=0.001), memory B cells (*P*=0.012), plasma cells (*P*=0.034), resting memory CD4^+^ T cells (*P*=0.051), Tregs (*P* < 0.001), and resting mast cells (*P*=0.002) were significantly increased in high-expression group. In contrast, activated memory CD4^+^ T cells (*P* < 0.001) and M1 macrophages (*P*=0.035) were significantly increased in low expression group. Moreover, the diverse TIIC subgroups presented a weak to moderate correlation (Figure 7(b)). Using TIMER, we also evaluated the correlation of EMID1 expression with immune infiltration levels. EMID1 was positively correlated with B cells and CD4^+^ T cells (Figure 7(c)).

## 4. Discussion

Lung cancer is the main cause of cancer-related deaths, with adenocarcinoma being the major subtype. To improve the prognosis of patients with LUAD, it is necessary to identify new biomarkers of LUAD [27]. The present study is the first to show that the expression of EMID1 is related to cancer and may be a prognostic biomarker of LUAD. The results revealed that low expression of EMID1 in LUAD was related to poor survival time and prognosis, as well as the progress of clinical pathology, such as late stage and metastasis of lung cancer. The study deployed GSEA to further explore EMID1 functions in LUAD and specified the following as differentially enriched in its high expression phenotype: melanogenesis, basal cell carcinoma, vasoconstriction, glycosaminoglycan biosynthesis of heparin sulfate, Notch signaling pathway, neuroactive ligand-receptor interaction, Hedgehog signaling pathway, ganglioside biosynthesis series, GnRH signaling pathway, and dilated heart myopathy. We also evaluated the relationship between EMID1 expression and level of immune infiltration in LUAD by CIBERSORT. The expression of EMID1 influences a variety of immune cells. All these results suggested that EMID1 might be an independent prognostic marker of LUAD.

Notch signaling pathway is involved in cell proliferation, differentiation, and survival, and is one of the common signaling pathways in cancer. Notch-activated mutations and amplification of Notch pathway play a key role in the progression of cancer [29]. It is a highly conserved ligand-receptor signaling pathway, which contains four Notch receptors and five ligands. The four receptors are Notch 1, Notch 2, Notch 3, and Notch 4, which have similar structures [30–32]. Anja Baumgart et al. found that lack of Notch 1 led to a reduction of early tumor formation, suggesting that Notch 1 plays a role in promoting cancer. However, the expression of Notch 2 receptor in NSCLC is weak, suggesting that Notch 2 may play an anticancer role in NSCLC [33]. Compared with Notch 1 and 2, Notch 3 receptor has received less attention, but its role cannot be ignored. Min Zhou et al. showed that activation of Notch 3 can promote the development of lung cancer, suggesting that Notch 3 may be a carcinogen of lung cancer [34]. Therefore, we speculated that increased expression of EMID1 might play an anticancer role by inhibiting the activity of Notch 1 and Notch 3, or by stimulating the activity of Notch 2. In all, through the study of biological functions, we can further understand the functions of EMID1.

Tumor-infiltrating lymphocytes, as a primary prognostic biomarker of tumor progression, can also serve to independently predict sentinel lymph node status and cancer survival [35, 36]. A significant aspect of our study entailed EMID1 expression with reference to immune infiltration levels in LAUD and concluded a positive correlation with B cells, thereby indicating that EMID1 regulated tumor immunology. Increasing evidence suggests that tumor-infiltrating B cells correlate with positive clinical outcomes in several cancers, producing antibodies whilst also acting as antigen-presenting cells (APCs) that intrinsically regulate cellular immunity in TME [37–39]. Moreover, B cells have the opposite effect on tumor immunity and progression, for example, B cells regulate adaptive immunity by releasing circulating cytokines or chemokines, thereby recruiting immunosuppressive myeloid cells, which eventually lead to chronic inflammation or neonatal cancer [40]. Hao et al. also correlated B cell infiltration with anti-PD-L1 therapy to potentially advance prospective treatment options for patients with lung cancer [41]. However, the mechanism of EMID1 regulating tumor-infiltrating B cells is not clear and additional research is needed.

### 4.1. Limitations

Our study has several shortcomings. (1) The clinical data types of our samples were less, which inevitably led to the loss of some useful information. (2) Our study did not analyze a signal mechanism at the cytological level. (3) This study did not carry out protein level analysis because there was not enough clinical sample data. Altogether, our conclusions require validation via an expanded clinical sampling in future research.

## 5. Conclusion

In all, our study assessed the relationship of EMID1 with clinicopathologic variables and survival outcomes and explored the mechanism of EMID1 in LUAD. Notch signaling pathway may be the main regulatory pathway of EMID1 in LUAD. In addition, the change in EMID1 expression was related to the proportion of B cells in LUAD, and EMID1 may play an important role in the immune environment of LUAD. Therefore, EMID1 may be a promising prognostic marker of LUAD.

## Figures and Tables

**Table 1:** Gene sets enriched in the high EMID1 expression phenotype.

Gene set name	NES	Adj.*P*	FDR
KEGG_BASAL_CELL_CARCINOMA	2.06	0.00	0.06
KEGG_MELANOGENESIS	2.05	0.00	0.04
KEGG_VASCULAR_SMOOTH_MUSCLE_CONTRACTION	1.96	0.00	0.07
KEGG_GLYCOSAMINOGLYCAN_BIOSYNTHESIS_HEPARAN_SULFATE	1.90	0.00	0.10
KEGG_NOTCH_SIGNALING_PATHWAY	1.83	0.01	0.16
KEGG_NEUROACTIVE_LIGAND_RECEPTOR_INTERACTION	1.83	0.00	0.14
KEGG_HEDGEHOG_SIGNALING_PATHWAY	1.80	0.00	0.16
KEGG_GLYCOSPHINGOLIPID_BIOSYNTHESIS_GANGLIO_SERIES	1.79	0.01	0.15
KEGG_GNRH_SIGNALING_PATHWAY	1.78	0.00	0.15
KEGG_DILATED_CARDIOMYOPATHY	1.72	0.01	0.21

NES: normalized enrichment score; NOM: nominal; FDR: false discovery rate. Gene sets with adj.*P*-value <0.05 and FDR <0.25 were considered as significantly enriched.

**Table 2:** The characteristics of lung adenocarcinoma patients.

Clinical characteristics		TCGA (*N* = 522)	%	GEO (*N* = 61)	%
Age (mean ± SD)		65.33 ± 10.02		59.41 ± 10.41	
Survival time (y)		2.11 ± 2.28		2.91 ± 2.44	

Gender	Female	280	53.60	28	45.90
	Male	242	46.40	33	54.10

Stage	I	279	53.40		
	II	124	23.80		
	III	85	16.30		
	IV	26	5.00		

Tumor status	T1	172	34.30		
	T2	281	53.80		
	T3	47	9.00		
	T4	19	3.60		

Lymph node	N0	335	64.20		
	N1	98	18.70		
	N2	75	14.40		
	N3	2	0.40		

Distant metastasis	M0	353	67.60		
	M1	25	4.80		

**Table 3:** EMID1 expression associated with clinical pathological characteristics (logistic regression).

Clinical characteristics	Total (N)	Odds ratio in EMID1 expression	*P*-value
Gender (Male VS female)	522	0.66 (0.46,0.93)	0.019
Age ( ≥ 65 VS < 65)	522	0.75 (0.52,1.06)	0.105
Stage (II. VS I)	514	0.83 (0.54,1.27)	0.388
Stage (III. VS I)	514	0.52 (0.32,0.86)	0.012
Stage (IV. VS I)	514	0.30 (0.11,0.71)	0.009
Tumor status (T2 vs. T1)	519	0.79 (0.54,1.17)	0.242
Tumor status (T3 vs. T1)	519	0.50 (0.25,0.96)	0.041
Tumor status (T4 vs. T1)	519	0.47 (0.17,1.23)	0.132
Distant metastasis (M1 vs. M0)	378	0.37 (0.14,0.87)	0.030
Lymph node (N1 vs. N0)	510	0.92 (0.58,1.45)	0.705
Lymph node (N2 vs. N0)	510	0.49 (0.28,0.81)	0.007

**Table 4:** Univariate and multivariate regression analysis of TCGA-LUAD.

Characteristics	Univariate analysis		Multivariate analysis	
	HR(95% CI)	*P* Value	HR(95% CI)	*P* Value
*T Stage*				
T1	Reference			
T2	1.521 (1.068–2.166)	0.020	1.401 (0.859–2.284)	0.177
T3	2.937 (1.746–4.941)	<0.001	3.378 (1.644–6.940)	<0.001
T4	3.326 (1.751–6.316)	<0.001	1.765 (0.690–4.517)	0.236

*N stage*				
N0	Reference			
N1	2.382 (1.695–3.346)	<0.001	1.853 (1.200–2.864)	0.005
N2&N3	2.968 (2.040–4.318)	<0.001	1.877 (0.728–4.844)	0.193

*M Stage*				
M0	Reference			
M1	2.136 (1.248–3.653)	0.006	1.002 (0.385–2.609)	0.996

*Gender*				
Female	Reference			
Male	1.070 (0.803–1.426)	0.642		

Age				
≤65	Reference			
>65	1.223 (0.916–1.635)	0.172		

*Residual tumor*				
R0	Reference			
R1&R2	3.879 (2.169–6.936)	<0.001	2.450 (1.131–5.308)	0.023

*Smoker*				
No	Reference			
Yes	0.894 (0.592–1.348)	0.591		

*Pathologic stage*				
Stage I&Stage II	Reference			
Stage III&Stage IV	2.664 (1.960–3.621)	<0.001	1.608 (0.607–4.255)	0.339

## Data Availability

All data are included in the article.
